# Deeper Spatial Statistical Insights into Small Geographic Area Data Uncertainty

**DOI:** 10.3390/ijerph18010231

**Published:** 2020-12-30

**Authors:** Daniel A. Griffith, Yongwan Chun, Monghyeon Lee

**Affiliations:** 1 School of Economic, Political and Policy Sciences, The University of Texas at Dallas, 800 West Campbell Road, Richardson, TX 75080, USA; ywchun@utdallas.edu; 2 Memory Business Division, Samsung Electronics Co. Ltd., 1, Samsungjeonja-ro, Hwaseong-si, Gyeonggi-do 18448, Korea

**Keywords:** big data, big spatial data, cancer, small area, small geographic area

## Abstract

Small areas refer to small geographic areas, a more literal meaning of the phrase, as well as small domains (e.g., small sub-populations), a more figurative meaning of the phrase. With post-stratification, even with big data, either case can encounter the problem of small local sample sizes, which tend to inflate local uncertainty and undermine otherwise sound statistical analyses. This condition is the opposite of that afflicting statistical significance in the context of big data. These two definitions can also occur jointly, such as during the standardization of data: small geographic units may contain small populations, which in turn have small counts in various age cohorts. Accordingly, big spatial data can become not-so-big spatial data after post-stratification by geography and, for example, by age cohorts. This situation can be ameliorated to some degree by the large volume of and high velocity of big spatial data. However, the variety of any big spatial data may well exacerbate this situation, compromising veracity in terms of bias, noise, and abnormalities in these data. The purpose of this paper is to establish deeper insights into big spatial data with regard to their uncertainty through one of the hallmarks of georeferenced data, namely spatial autocorrelation, coupled with small geographic areas. Impacts of interest concern the nature, degree, and mixture of spatial autocorrelation. The cancer data employed (from Florida for 2001–2010) represent a data category that is beginning to enter the realm of big spatial data; its volume, velocity, and variety are increasing through the widespread use of digital medical records.

## 1. Introduction

Currently popular scientific terms include “big data” and “big spatial data.” Especially when dealing with medical and public health data, one big (spatial) data feature meriting more attention is (geographic) resolution. This feature interfaces with the Law of Large Numbers (LLN), a statistical principle that may be summarized as follows:

Given random sampling, as a sample size, *n*, goes to infinity, the empirical probability of an event approaches its theoretical probability (given by its probability mass or density function): the distribution of a random sample tends to resemble the distribution for its parent population more closely as n increases.

In other words, certain statistics computed from a sample tend toward their corresponding parameter values as *n* increases, which relates the LLN to the Central Limit Theorem (CLT), another fundamental principle of statistics (see [1]). These two statistical concepts interface with interplay between the notions of big data and of resolution, with the latter sometimes moderating the former. This theme constitutes the topic of this paper.

The phrase big (spatial) data refers to extremely large datasets, with the meaning of “big” remaining ambiguous, and not necessarily referring to amount. Rather, the following selected data properties constitute the differentiating features: volume (i.e., quantity), velocity (i.e., availability speed), variety (i.e., diversity of types), variability (i.e., information content meaning constantly changing), veracity (i.e., degree of reliability/accuracy), and complexity (i.e., structured, semi-structured, quasi-structured, and unstructured). Big data are burdened with the following requisites, which need to be performed efficiently and effectively: analyzing, capturing, privacy preserving, querying, sharing, storing, transferring, updating, and visualizing [2]. These are the same handling requirements that distinguish between geographic information system datasets and many other types of data [3] (p. 1), furnishing a strong link between the notions of “big data” and “big spatial data.” Cressie et al. [4] (p. 115) note that “… the sheer size of a massive [dataset] may challenge and, ultimately, defeat a statistical methodology that was designed for smaller [datasets] …” One such failure is statistical significance testing: with a large enough dataset, virtually all results are statistically significant. Conversely, with a small enough dataset, virtually no results are statistically significant (i.e., small sample sizes undercut the trustworthiness of statistical inferences, with a sample size of one, in and of itself, unable to furnish any information about the precision of its sample statistic; see [5]). Another stems from such data almost always being non-random, such that without adequate data analytic precautions, resulting correlations can be spurious, predictions can be erroneous, and results can be unsatisfactory.

With these aforementioned caveats in mind, the purpose of this paper is to establish some deeper insights into big spatial data, with special reference to public health data, in terms of their uncertainty through one of the hallmarks of georeferenced data, namely spatial autocorrelation (SA), coupled with small geographic areas (re. resolution). A focal point is the intersection of SA with the issue of instability of estimates in small sample sizes, and/or over small geographic areas in the presence of what appears to be big spatial data. Impacts of interest concern the nature, degree, and mixture of SA. Big data analyses focus on hypothesis generation rather than hypothesis testing [6], and hence one important theme for big spatial data is relationship stability, especially with regard to heterogeneity, across geography (as well as time). Accordingly, this paper studies six Florida metropolitan statistical areas (MSAs) to address this geographic stability aspect. Meanwhile, big healthcare data (increasingly acquired from electronic health records) are not only complex, but also have unique characteristics, beyond their large size (which often is relative to the usually unavoidable extremely small clinical trial sample sizes; [7]), that both facilitate and complicate the uncovering of insights about an observable public health phenomenon. To this end, this paper studies selected cancer cases for the period 2001–2010. Its aim is to identify and assess geographical patterns within the context of SA to establish a better understanding of small geographic area data uncertainty [i.e., the instability of small sample size (à la the CLT) and/or small geographic area estimates].

### 1.1. A Motivating Example: The Role of Resolution

Geocoding of individuals allows for their post-stratification by areal units such as ZIP codes and census blocks, block groups, and tracts, these latter three polygon types being devised by the United States (US) Census Bureau [8]. These units constitute small areas. Aggregated socio-economic/demographic attribute data often are available for these geographic polygons, enabling data merging for observational studies involving ecological correlation analysis. This data analytic framework often suffers from post-stratification defects, especially when it yields small geographic area sample sizes. Spielman et al. [9], after studying US census data uncertainty causes, show that these data tend to have higher margins of error for smaller geographic areal units. In other words, resolution matters.

**Figure 1:**
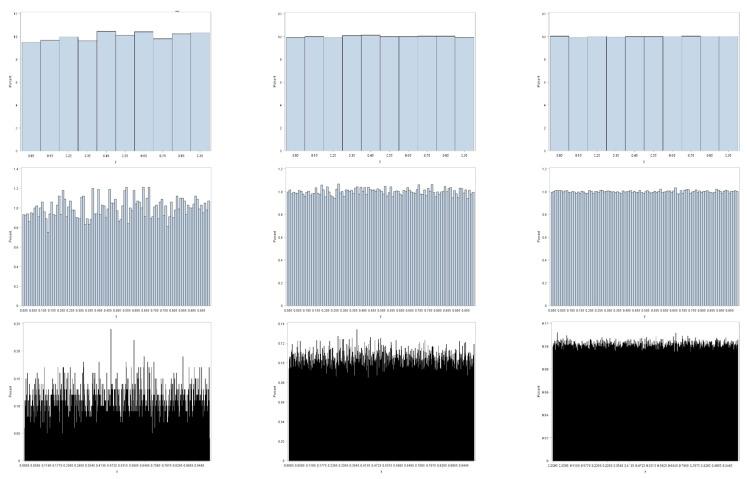
Histograms for random samples from a continuous uniform distribution. Sample size *n*: left—10,000; middle—100,000; right—1,000,000. Bin size: top—0.1; middle—0.01; bottom—0.001.

Figure 1 illustrates this preceding contention, furnishing an appropriate example here, because binning of observed values to construct histograms parallels the geographic aggregation of geocoded points into areal unit polygons. This illustration employs three random sample sizes: 10^4^, 10^5^, and 10^6^. It also employs three resolutions (i.e., bin sizes): 0.1, 0.01, and 0.001. Sampling is from a uniform distribution; the LLN implies that as *n* increases these histograms should converge on their parent theoretical uniform frequency distribution for the interval [0, 1]. All three coarser resolutions display little deviation from a uniform distribution, with this deviation decreasing with increasing sample size. As resolution becomes finer, the *n* = 10^4^ sample size fails to display a close correspondence with its parent uniform distribution: the moderate resolution has noticeable variation, and the fine resolution has conspicuous variation in bin frequencies. These deviations dampen out as *n* increases to 10^5^, and then to 10^6^. However, if the bin size were decreased to 0.0001 for the *n* = 10^6^ sample size, then it, too, would exhibit obvious deviations from a uniform distribution. One principal implication is that small area resolution, both geographic and non-geographic, plays a critical role in determining the meaning of the notion of big data, particularly with regard to its volume and variability properties.

### 1.2. Effective Geographic Sample Size: A Complicating Factor

One of the complexities of spatial data arises from their being correlated data containing redundant or duplicate information (i.e., they are spatially autocorrelated; [10]). The SA latent in most geographically distributed socio-economic/demographic data is positive, and roughly ranges from 0.4 to 0.6 for provincial/state, county, and census tract resolutions across national and regional geographic landscapes studied to date. The SA latent in most remotely sensed images also is positive, and roughly ranges from 0.9 to 0.99, certainly for a 30 m-by-30 m pixel size (e.g., Landsat images). The effective geographic sample size for *n* areal units is the number, n*, of equivalent independent and identically distributed observations based upon the nonredundant information content in a given dataset [11,12,13,14,15]; n*, like degrees of freedom, may not be an integer. 

Table 1 furnishes examples of *n* and n* that have been gleaned from the literature. The calculation of n* is somewhat sensitive to the assumed spatial statistical model. Nevertheless, even with moderate positive SA (PSA), substantial reductions in effective sample size occur. Reductions for remotely sensed images potentially could decrease from an extremely large *n* to an n* < 30.

### 1.3. The Florida Cancer Dataset

**Figure 2:**
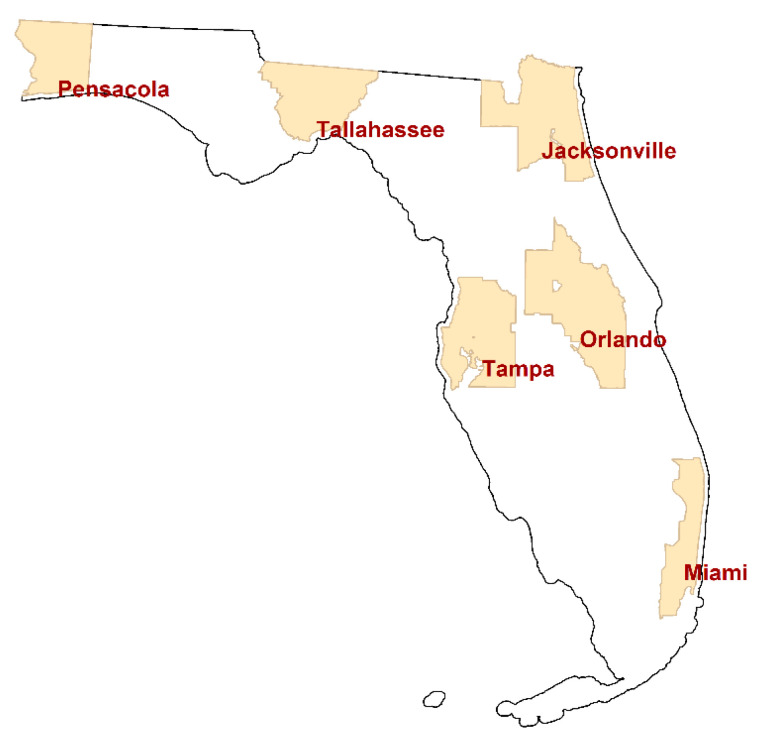
The State of Florida, and the locations of the six studied metropolitan statistical areas (MSAs).

This paper summarizes analyses of individual cancer cases located in the following six Florida MSAs: Jacksonville, Miami, Orlando, Pensacola, Tallahassee, and Tampa. Figure 2 portrays the location of these MSAs, which furnish a wide geographic coverage of the state. This study utilizes six different cancer types that have a relatively large number of cases: breast, female breast, colorectal, lung & bronchus, melanoma skin, and urinary bladder. The other counties have relatively small numbers of cancer cases, so that a considerable number of small areal units in the counties (e.g., census block groups) have zero cases even for these more common cancer types. Hence, this study focuses on the six counties.

Individual cancer patient data in Florida from 2001 to 2010 were obtained from the Florida Cancer Registry of the Florida Department of Health and then analyzed (with rigorous University of Texas at Dallas and Florida Department of Health Institutional Review Board monitoring and approval). This dataset contains limited individual patient demographic characteristics, such as age and gender, as well as residential locations in the form of geocoded x, y coordinates. These data includ no information that can reveal patient identities. Cancer patient points that were inadequately geocoded using home address matching were removed from the dataset as part of its data cleaning (The Florida Department of Health contracts address matched to a private vendor that uses proprietary geocoding software. Authors’ data cleaning resulted in a dataset with a geocoding success rate of roughly 90%). These points were geocoded either to a ZIP code centroid with a partial address (i.e., ZIP code only), or were assigned to areal units that have zero population in both the 2000 and 2010 US decennial census reports. Duplicate registry entries were also removed. After this data cleaning exercise, 9,444,852 records remained for use in this study. Table 2 presents the number of cancer cases from this reduced set of records for the six cancer types in the individual MSAs.

Geographically aggregated cancer cases were converted to rates per 100,000 population, in part, to adjust for the varying sizes of the areal units (i.e., census block groups). Other analyses of these data include articles by Hu et al. [17,18] and Lee et al. [19,20], which furnish additional details about these data.

## 2. Standardized Cancer Rates

Populations tend to be heterogeneous, and hence can be subdivided into more homogeneous sub-populations. The goal of standardization is to adjust for this heterogeneity in order to establish measures that are comparable across the sub-populations (e.g., cohorts) differing in, for example, age and/or other demographic characteristics (e.g., sex). Ignoring this heterogeneity results in crude rates, measures that may be distorted because the sub-populations differ in size, and hence can differentially influence these measures. One approach to incorporating a reasonable weighting of the various sub-populations is to establish a standard, a reference population with a particular composition. The resulting standardized measure is the summary rate that would be observed in a population with the specified composition [21]. In other words, standardization is an indirect method that adjusts for confounding factors, such as age, to remove their distorting effects from population comparisons.

Two demographic factors impacting cancer rates are age and sex. The statistical small area problem here is the cross-tabulation of age and sex. With regard to resolution, these cross-classification cells are the bins to be filled by a particular set of geocoded cancer data. The following three reference populations may be considered: World, US, and Florida (FL). The formula quantifying this measure may be written as follows:

(1) $$R_{i}=\sum_{h}^{H}\sum_{k}^{K}\frac{C_{\mathrm{hki}}}{P_{\mathrm{hki}}}10^{5}\frac{P_{\mathrm{hk}}^{*}}{\sum_{h}^{H}\sum_{k}^{K}P_{\mathrm{hk}}^{*}}=\frac{\sum_{h}^{H}\sum_{k}^{K}C_{\mathrm{hki}}\frac{P_{\mathrm{hk}}^{*}}{P_{\mathrm{hki}}}}{\sum_{h}^{H}\sum_{k}^{K}P_{\mathrm{hk}}^{*}}10^{5}=\sum_{h}^{H}\sum_{k}^{K}\frac{C_{\mathrm{hki}}}{P_{\mathrm{hki}}}\frac{P_{\mathrm{hk}}^{*}}{\sum_{h}^{H}\sum_{k}^{K}P_{\mathrm{hk}}^{*}}10^{5}$$

where subscript h denotes each of H age groups, subscript k denotes each of K sex groups, $C_{\mathrm{hki}}$ denotes the number of cancer cases in cross-classification h-k in areal unit i, $P_{\mathrm{hki}}$ denotes the population count in cross-classification h-k in areal unit i—$\left(C_{\mathrm{hki}}/P_{\mathrm{hki}}\right)\times 10^{5}$ is the crude rate per 100,000—and $P_{\mathrm{hk}}^{*}$ denotes the population count in cross-classification h-k in the reference population used for standardization purposes.

### 2.1. Some Simple Comparisons of the Reference Populations

**Figure 3:**
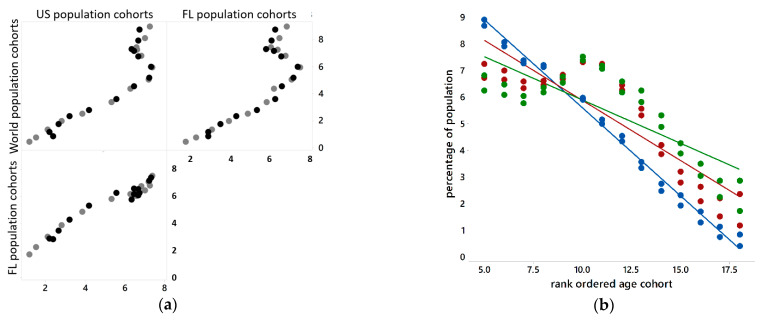
Scatterplots of reference population distributions, ages 20+. Left (**a**): paired by age cohorts; black denotes male, grey denotes female. Right (**b**): ordered by age cohorts; blue denotes World, red denotes US, and green denotes Florida (FL).

The reference populations have different distributions across 18 5-year age cohorts (Figure 3a), with the last cohort being 85+. Percentages by age cohort vary more for very young people, and tend to decrease as people get older (Figure 3b). Figure 3 suggests that age- and sex-standardized results should be similar across the three reference populations. It also suggests that older age cohorts are rarer events, and hence may constitute more problematic data points vis-à-vis the LLN.

### 2.2. Some Comparisons of the Crude and Standardized Rates

Table 2 reveals that: (a) an aggregation of the six cancer types studied here results in 9,444,852 case occurrences; and the number of age-sex-block group cross-classifications here is 1,038,100. Table 3 reveals that roughly 90% of these cross-classifications have zero entries. This situation is far more extreme than that portrayed in Figure 1, which is based upon random allocations to cross-classification subgroups. Often big spatial data do not constitute a random sample. Table 2 documents that all six cancer types are present in each of the Florida MSAs. Table 3 documents that few block groups have no cancer cases for each of these cancer types. The primary culprit here is the distribution of cases across age cohorts.

**Figure 4:**
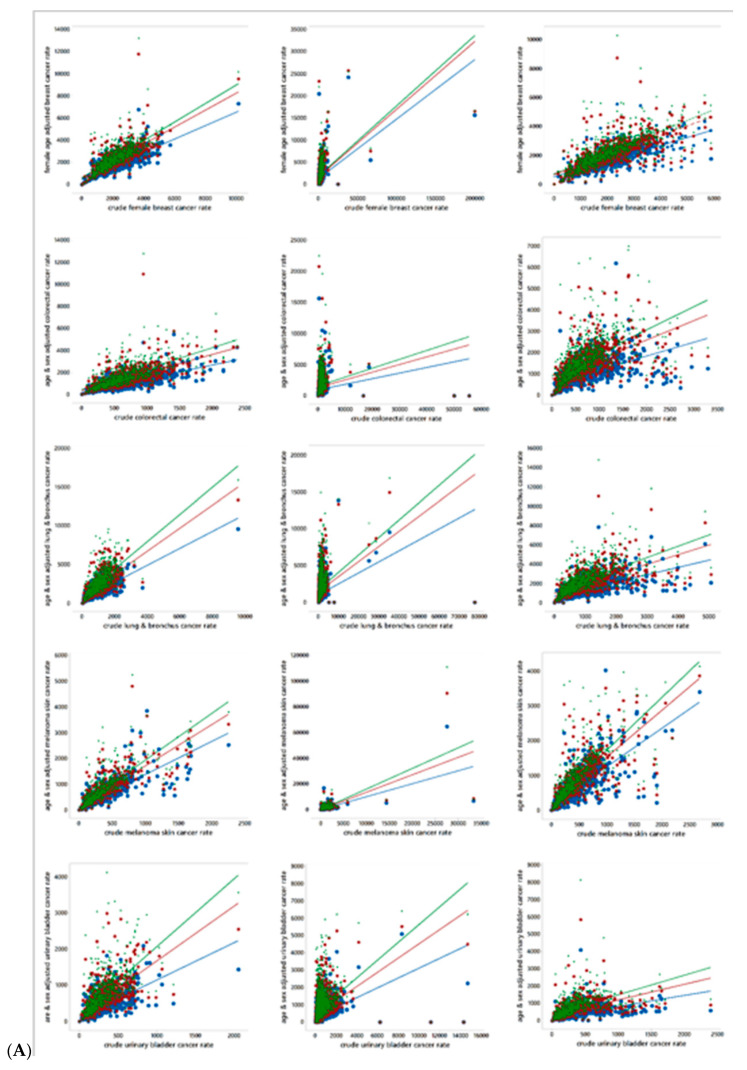

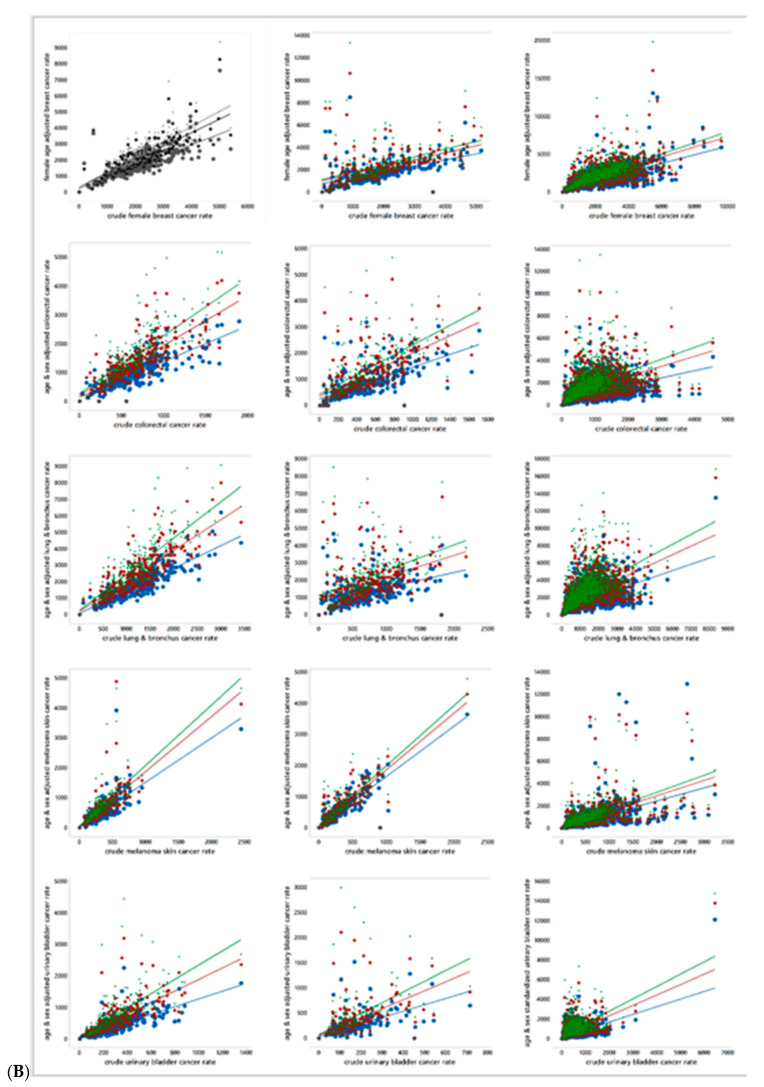
Scatterplots of crude cancer rates (vertical axis) versus age-and sex-adjusted cancer rates (horizontal axis); blue denotes World, red denotes US, and green denotes Florida (FL). (**A**). Top to bottom: cancer type (female breast, colorectal, lung & bronchus, melanoma skin, urinary bladder). Left to right: MSA (Jacksonville, Miami, Orland). (**B**). Top to bottom: cancer type (female breast, colorectal, lung & bronchus, melanoma skin, urinary bladder). Left to right: MSA Pensacola, Tallahassee, Tampa).

Figure 4 presents scatterplots for the associations between age-and-sex standardized together with crude cancer rates, by cancer type and MSA. Each scatterplot includes three age-sex adjustments, one based upon the World, one based upon the US, and one based upon the state of Florida reference population distributions. The trend lines appear in this rank order in all scatterplots. Equation (1) discloses that standardization modifies a crude cancer count by inflating it, deflating it, or leaving it unchanged in accordance with the ratio of the block group and reference population cohort counts. The scatterplots appearing in Figure 4 highlight that these adjustments can create leverage and influential points affecting their corresponding trend line. Meanwhile, Table 3 reports extreme outliers and the frequency of block groups with zero rates. These two heavy tail indicators imply that few of these rates can approximate a normal random variable by being subjected to a Box-Cox power transformation. Such power transformations tend to be successful when deviation occurs in only one tail, rather than two tails. Furthermore, large numbers of zeros defy tail stretching. In other words, the nature of these data suggests that they need to be analyzed using non-normal probability models, which, in turn, require the rates to be rounded to integers. This rounding introduces additional uncertainty into an analysis, which should be much less than the specification error that would be introduced by assuming a normal approximation when this approximation is very poor. Here this rounding error increases/decreases (7 increases, and 13 decreases) the variance of the rates by less than 0.01%.

Table 4 furnishes popular SA measures, namely the Moran Coefficient (MC) and the Geary Ratio (GR), for the geographic distributions of the five cancer types within each of the six MSAs. These measures indicate that the levels of SA vary by cancer type, have some trends across the MSAs, and are dramatically impacted by age-sex standardization. They also indicate that, for the most part, the prevailing SA essentially is weak and positive.

## 3. Spatial Autocorrelation and Public Health Data

SA is a feature of georeferenced health data; Jacquez [22] summarizes a number of sources. Disease mapping reveals that cases tend to cluster in geographic space, often forming hotspots and coldspots. This outcome may result from a disease being contagious, or from exposure to some common underlying environmental factor (e.g., a geographically concentrated contaminant) that couples with genetic susceptibility to promote occurrences of an illness. Furthermore, Schelling’s [23,24] work highlights that neighborhoods tend to house people with many similar lifestyle characteristics, such as income and density of housing, rather than random mixtures of people. These contexts imply the presence of PSA. One consequence of the resulting SA is overdispersion in count data described by binomial, negative binomial, and Poisson probability models.

However, cancer is not contagious; it may link to a common exposure, and often takes a goodly number of years to develop, introducing noise that may well mask links. Consequently, one may expect geographic distributions of cancer rates to be characterized by weak PSA [25]. However, it may also exhibit a negative SA (NSA) component, which may be specific to the census block group geographic resolution. One source of this NSA in MSAs is attributable to land use zoning practices, which can juxtapose zero and non-zero population count areal units. Another is local social network structures and screening rates: if a person gets screened, s/he may encourage his/her neighbors to get screened, especially if the screening results in a cancer diagnosis. One possible outcome is a cancer map with adjacent high and low rates. This same type of outcome can arise from targeting specific neighborhoods for screening, which again would produce such local contrasts.

### 3.1. Moran Eigenvector Spatial Filtering: A Brief Overview

Moran eigenvector spatial filtering [26] is a novel spatial statistical methodology addressing SA that adds a set of synthetic proxy variables, which are eigenvectors extracted from a doubly centered version of an *n*-by-*n*, usually binary 0–1, spatial weights matrix **C** that links geographic objects together in space as control variables to filter SA out of residuals and transfer it to the mean response in a regression model specification (this modification creates a spatially varying intercept term). These control variables identify and isolate the stochastic spatial dependencies among georeferenced observations, thus allowing model parameter estimation to proceed with observations mimicking being independent.

The crucial matrix **C** mathematical attributes are eigenfunctions, which are *n* pairs of *n*-tuples and scalar quantities computed via the matrix determinant of a modified version of matrix **C**, **MCM**, where $M=\left(I-11^{T}/n\right)$, **I** denotes the *n*-by-*n* identity matrix, and **1** denotes the *n*-by-1 vector of ones: a scalar (eigenvalue) and a vector (its corresponding eigenvector). Eigenvalues are the *n* scalar solutions to the *n*^th^ order polynomial matrix determinant equation det (**MCM** − λ**I**) = 0; the corresponding eigenvectors **E** are the non-trivial vector solutions to the equation (**MCM** − λ**I**)**E** = **0**. These eigenfunctions are the basis of Moran eigenvector spatial filtering (MESF), and are the synthetic variates that account for nonzero SA in spatial regression residuals.

The MC index of SA may be written, using matrix notation, for some random variable Y with *n* georeferenced observations, as

(2) $$\frac{n}{1^{T}C1}\frac{Y^{T}(I-11^{T}/n)C(I-11^{T}/n)Y}{Y^{T}(I-11^{T}/n)Y}=\frac{n}{1^{T}C1}\frac{Y^{T}MCMY}{Y^{T}MY}$$

ESFs are constructed as linear combinations of the **MCM** matrix eigenvectors. Appealing properties of these eigenvectors include: (1) they are mutually orthogonal and uncorrelated; (2) one vector is proportional to **1**, the intercept covariate in a regression model; and (3) eigenvalues index, and eigenvectors support the visualizing of, various distinct natures and degrees of SA.

Including eigenvectors as covariates, and selecting relevant ones with a stepwise procedure, enables SA to be accounted for in a conventional statistical estimation context, in either a linear or a generalized linear model (GLM) specification. In many GLM applications, SA tends to account for about half of any detected overdispersion.

### 3.2. Spatial Autocorrelation and Big Spatial Data

Big spatial data can take on various forms. One is an increase in the number of areal units, which relates more to infill than to increasing domain asymptotics in spatial sampling, but relates mostly to data volume. Recent asymptotic analyses [27,28] reveal that across a wide range of random variable types, sample sizes, and geographic surface partitionings, the MC outperforms the GR as an index of SA. This finding bolsters the conceptual basis of MESF.

Another form is moving from very small sample sizes typical of many clinical trials (Institute of Medicine, 2001) to millions of cases gleaned from medical records. The number of such cases in this study, for five specific cancer types, is nearly 9.5 million. But post-stratification, for both standardization and spatial analysis purposes, moves these data away from the big spatial data realm, at least to some degree. The SA latent in them appears to be a mixture of PSA and NSA. MESF is a methodology particularly suitable for analyzing this SA mixture. Because MESF involves *n* eigenvectors, and *n* can be relatively large for MSAs (e.g., 3377 for Miami), implementing MESF becomes a challenge here, particularly when coupled with a GLM and stepwise eigenvector selection. The number of required estimation iterations at each eigenvector selection step for a model specification, and the substantial size of a candidate eigenvector set (which now has vectors representing both PSA and NSA), challenges estimation algorithms that successfully work for smaller georeferenced datasets, such as the one for Tallahassee (*n* = 233). In other words, nonlinear estimation combined with combinatorics can magnify a moderate-to-large spatial data problem into a big spatial data problem.

### 3.3. Constructing ESFs for Florida MSA Standardized Cancer Rates

Section 2.2 presents an argument for rejecting the use of a normal approximation when conducting a spatial analysis of standardized cancer rates (e.g., the presence of zeroes, outliers, and leverage observations). The analysis summarized in this section employs a Poisson probability model because researchers most often employ it to describe vital statistics rates. The assumption of a Poisson random variable requires rounding of the standardized rates to integers, the form of their crude rate counterparts (the noise introduced by this arithmetic operation appears to be trivial; see Section 2.2). Meanwhile, equation (1) suggests several possible offset variables, including not positing one (a rate per 100,000 results in a constant across all cancer types and MSAs, and simply modifies each intercept by ${\mathrm{LN}}_{10}\left(10^{-5}\right)=-5$). Given the presence of overdispersion, this Poisson assumption is replaced with a negative binomial random variable assumption.

The inferential basis for the cancer data analysis summarized in this paper is model based; cancer cases are not random samples. As such, acceptable diagnostics need to accompany the spatial statistical models employed in this analysis. The presence of overdispersion (i.e., extra-Poisson variation) and of SA are two important data features needing to be accounted for in order to satisfy important model properties. Overdispersion relates to uncertainty, primarily through noise and abnormalities in data. Here minimizing specification error helps to address these data features: a normal approximation was replaced by a Poisson specification (involving rounding of rate numerators), which then was replace with a negative binomial specification (to account for variation described by *σ*^2^ = *μ*(1 + *ημ*) rather than simply *σ*^2^ = *μ*, where *η* > 0 denotes the dispersion parameter). The outcome of this sequence of substitutions should be, for example, a deviance statistic essentially equal to 1. Overdispersion can relate to a random effects term. As such, latent SA links to a spatially structured component of this term, whereas the residual dispersion parameter links to a spatially unstructured component of this term.

Because an auto-negative binomial model can capture only NSA, and most georeferenced phenomena display PSA, the analysis summarized here employed a MESF negative binomial (MESFNB) model specification. Table 5 summarizes the number of modified spatial weights matrix eigenvectors in a given MSA candidate set; because the expectation is a mixture of PSA and NSA, Table 5 contains counts for both SA natures.

Table 6 summarizes MESFNB estimation results. A simulation experiment was conducted for each sample size, sampling the mean for each of 10,000 replications from a gamma distribution with randomly selected parameters from the interval (0, 10). Extreme deviance results based upon this experiment indicate that only melanoma skin and urinary bladder cancer in Jacksonville have dispersion parameters statistically significantly greater than what is expected for the NB specification employed here. Meanwhile, the level of SA in these data is low. Roughly 70% of the geographic distributions of studied cancers have a mixture of PSA and NSA in which the PSA component dominates. Urinary bladder cancer is the only cancer type that consistently has a mixture in which NSA dominates. Five geographic distributions have only a PSA component. Urinary bladder cancer in Tallahassee fails to exhibit any SA. Of note is that the age-sex standardization transformation appears to have introduced noise into these georeferenced data, resulting in a shrinkage of their SA index values toward zero SA.

## 4. Conclusions

The analyses summarized in this paper emphasize that SA latent in cancer data appears to be weak and a mixture of PSA and NSA. Both this feature and the uncovered extra-Poisson variation imply the need for a spatially structured and a spatial unstructured random effects term in a model specification. These components should serve as clues for selecting substantive covariates to include in a MESFNB model specification. They also may relate to the age-sex standardization transformation used; just as with Box-Cox and Box-Tidwell power transformations, perhaps such a standardization needs to be applied to both sides of the equation.

The data analyzed for this paper comprises nearly 9,500,000 cancer cases, which is big spatial data based upon most sample sizes used for clinical trials, or for medical panel surveys (which often involve thousands or more). However, with post-stratification, even with big data, a researcher can encounter the problem of small local sample sizes materializing, which tends to inflate local uncertainty and undermine otherwise sound statistical analyses. Accordingly, georeferenced data analyses must address the resulting bias, noise, and abnormalities in these data.

This paper’s georeferenced cancer data spatial analyses fulfill its aim, namely to identify and assess geographical patterns within the context of SA, rendering a better understanding of small geographic area data uncertainty. SA obscures effective sample size, impacts the efficacy of the LLN and the CLT, differs between crude and standardized rates, and meshes with geographic resolution, introducing instabilities into spatial statistical estimates. Various parts of this paper illustrate these contentions.

Finally, this paper summarizes findings about the nature, degree, and mixture of SA in selected geographic distributions of cancer. One useful finding here is that a mixture of PSA and NSA is the norm for cancer data across six different MSAs [also see 17]. One useful future research theme concerns whether or not the polygon-based SA studied in this paper holds for noncontiguous geographic areas, given the point nature of the individual cancer cases.

One important outcome of this work is that this paper furnishes deeper spatial statistical insights into small geographic area data uncertainty. Small sample and empty cross-classification cells complicate post-stratified data analyses. A mixture of PSA and NSA complicates spatial data analyses. The presence of non-zero SA reduces the sample size (see Table 7). Data anomalies and excessive zeroes negate the validity of normal approximation analyses, increasing the numerical intensity of proper analyses, but with a MESFNB model specification furnishing a good description of the spatial distribution of standardized cancer rates at a reasonably fine geographic resolution.

## Figures and Tables

**Table 1:** Selected illustrative effective geographic sample sizes.

Geographic Landscape	Variable/Model	*n*	n*	Publication
Murray smelter site	lead (Pb)/SAR	253	77.0	[11]
	lead (Pb)/geostatistics		58.2	
	lead (Pb)/MESF		159.8	
Everglades	biomass/SAR	15,000,000	337,401	[16]
Adirondack	NDVI/SAR	257,033	1182	
Yellowstone	Factor 1/SAR	118,800	2236	
MESF denotes Moran eigenvector spatial filtering; SAR denotes simultaneous autoregressive model				

**Table 2:** Case counts for selected cancer types.

Variable		Jacksonville	Miami	Orlando	Pensacola	Tallahassee	Tampa
*n* (# block groups)		699	3377	833	267	233	2006
total # cases		987,796	4,166,052	1,567,398	332,549	272,146	2,122,641
breast: C50.0-C50.9	# cases	10,409	45,691	14,687	3559	2543	24,772
female breast: C50.0-C50.9	# cases	10,303	45,028	14,522	3520	2520	24,484
colorectal: C18.0-C18.9, C19.9, C20.9	# cases	5732	30,537	9147	2065	1207	17,001
lung & bronchus: C34.0-C34.9	# cases	9420	34,681	12,400	3755	1749	25,666
melanoma skin: C44.0-C44.9	# cases	2745	14,291	4803	934	675	8131
urinary bladder: C67.0-C67.9	# cases	2405	13,003	3727	894	274	7853

NOTE: International classification of diseases for oncology (ICD) codes follow cancer types; # denotes the number of cases or observations.

**Table 3:** Non-normal frequency distribution tail characteristics: zeroes and extreme large-value outliers.

Variable		Jacksonville	Miami	Orlando	Pensacola	Tallahassee	Tampa
*n* [# block groups (BGs)]		699	3377	833	267	233	2006
# 0 case BGs		0	23	0	2	4	5
% age-sex-BG 0 s		80.3	82.3	78.3	80.7	86.4	81.6
% age-sex-BG 0 s, 20–54 age cohort		90.1	92.1	89.3	91.0	92.4	92.1
breast: C50.0-C50.9	# 6-sigma outliers						
	# 0 case BGs	2	61	1	4	8	20
female breast: C50.0-C50.9	# 6-sigma outliers	1/2/2/2	3/8/8/8	0/0/1/1	0/1/1/1	0/1/0/1	1/5/4/5
	# 0 case BGs	2	62	1	4	8	22
colorectal: C18.0-C18.9, C19.9, C20.9	# 6-sigma outliers	0/1/1/1	5/9/9/5	0/1/0/0	0/0/0/0	0/0/0/0	1/4/4/5
	# 0 case BGs	11	105	11	3	19	57
lung & bronchus: C34.0-C34.9	# 6-sigma outliers	1/1/1/1	4/8/8/7	0/2/2/2	0/0/0/0	0/0/0/0	1/2/2/3
	# 0 case BGs	6	95	7	4	12	24
melanoma skin: C44.0-C44.9	# 6-sigma outliers	1/1/1/1	4/2/3/2	1/1/0/0	1/2/2/2	1/1/1/1	6/7/7/7
	# 0 case BGs	130	754	92	32	67	289
urinary bladder: C67.0-C67.9	# 6-sigma outliers	1/0/0/1	6/7/8/10	1/1/2/2	0/0/0/0	0/0/0/1	2/2/2/3
	# 0 case BGs	74	477	98	25	97	264

NOTE: use of the 6-sigma criterion emphasizes extreme outliers; entries are crude/world/US/ Florida standardized rates; International classification of diseases for oncology (ICD) codes follow cancer types; # denotes the number of cases or observations.

**Table 4:** Traditional SA indices for the five selected cancer types by MSA and standardization reference population.

Cancer	Crude		World Standardized		US Standardized		FL Standardized	
	MC	GR	MC	GR	MC	GR	MC	GR
*Jacksonville MSA*								
Female breast	0.16	0.79	0.11	0.84	0.10	0.83	0.10	0.83
Colorectal	0.17	0.80	0.10	0.84	0.08	0.86	0.08	0.86
Lung & bronchus	0.15	0.80	0.24	0.73	0.23	0.74	0.21	0.75
Melanoma skin	0.29	0.69	0.20	0.78	0.20	0.76	0.18	0.78
Urinary bladder	0.08	0.89	−0.02	0.98	−0.04	1.00	−0.04	1.00
*Miami*								
Female breast	0.03	0.95	0.08	0.94	0.08	0.95	0.07	0.94
Colorectal	0.03	0.87	0.06	0.99	0.06	0.98	0.06	0.96
Lung & bronchus	0.04	1.16	0.14	0.93	0.14	0.90	0.14	0.89
Melanoma skin	0.12	1.03	0.08	0.76	0.06	0.76	0.05	0.77
Urinary bladder	0.16	1.38	0.09	0.88	0.09	0.87	0.08	0.87
*Orlando*								
Female breast	0.25	0.72	0.17	0.80	0.18	0.80	0.18	0.79
Colorectal	0.29	0.64	0.09	0.88	0.08	0.90	0.08	0.90
Lung & bronchus	0.37	0.56	0.19	0.77	0.17	0.79	0.16	0.80
Melanoma skin	0.34	0.63	0.21	0.75	0.22	0.74	0.21	0.74
Urinary bladder	0.30	0.63	0.00	0.96	0.00	0.96	0.00	0.96
*Pensacola*								
Female breast	0.11	0.88	0.05	0.97	0.05	0.94	0.04	0.94
Colorectal	0.07	0.89	0.08	0.84	0.10	0.84	0.08	0.86
Lung & bronchus	0.14	0.84	0.21	0.76	0.22	0.74	0.21	0.73
Melanoma skin	0.12	0.77	0.03	1.04	0.02	1.02	0.01	0.98
Urinary bladder	0.07	0.96	0.00	0.90	-0.01	0.93	-0.02	0.93
*Tallahassee*								
Female breast	0.33	0.67	0.09	0.96	0.09	0.96	0.09	0.95
Colorectal	0.15	0.79	0.09	0.85	0.09	0.86	0.08	0.86
Lung & bronchus	0.14	0.79	0.05	0.90	0.05	0.92	0.04	0.92
Melanoma skin	0.35	0.74	0.26	0.82	0.27	0.82	0.26	0.83
Urinary bladder	0.07	0.84	0.01	0.93	0.01	0.93	0.01	0.93
*Tampa*								
Female breast	0.19	0.78	0.02	0.95	0.02	0.95	0.02	0.95
Colorectal	0.26	0.70	0.05	0.94	0.05	0.92	0.05	0.92
Lung & bronchus	0.33	0.65	0.17	0.78	0.16	0.80	0.14	0.82
Melanoma skin	0.37	0.58	0.11	1.03	0.15	0.92	0.16	0.88
Urinary bladder	0.26	0.69	0.03	0.86	0.03	0.86	0.04	0.87

**Table 5:** The size of the candidate eigenvector sets based upon $|{\mathrm{MC}}_{j}/{\mathrm{MC}}_{1}|\geq 0.25$.

Variable	Jacksonville	Miami	Orlando	Pensacola	Tallahassee	Tampa
*n* (# block groups)	699	3377	833	267	233	2006
# PSA eigenvectors	179	846	218	68	63	519
# NSA eigenvectors	254	1146	316	99	91	727

NOTE: # denotes the number of observations or eigenvectors.

**Table 6:** SA analysis of age-sex standardized cancer rates, world population as the reference.

Variable	Female Breast	Colorectal	Lung & Bronchus	Melanoma Skin	Urinary Bladder
*Jacksonville (n = 699)*					
# PSA eigenvectors	24	16	32	16	1
Pseudo-R^2^	0.18	0.14	0.30	0.17	0.01
# NSA eigenvectors	17	10	10		3
Marginal Pseudo-R^2^	0.10	0.06	0.06		0.04
Dispersion parameter	0.15	0.14	0.24	1.25	1.24
Deviance	1.13	1.18	1.18	**2.29**	**1.35**
*Miami (n = 3377)*					
# PSA eigenvectors	66	32	20	33	23
Pseudo-R^2^	0.12	0.07	0.08	0.03	0.04
# NSA eigenvectors	30	18	0	1	5
Marginal Pseudo-R^2^	0.05	0.05	0.00	0.00 ^a^	0.05
Dispersion parameter	0.41	0.62	0.82	3.01	1.82
Deviance	1.18	1.19	1.17	1.21	1.24
*Orlando (n = 833)*					
# PSA eigenvectors	45	24	47	16	3
Pseudo-R^2^	0.28	0.17	0.27	0.18	0.03
# NSA eigenvectors	42	26	20	5	4
Marginal Pseudo-R^2^	0.18	0.14	0.10	0.03	0.03
Dispersion parameter	0.13	0.28	0.25	1.43	1.48
Deviance	1.16	1.20	1.20	1.26	1.24
*Pensacola (n = 267)*					
# PSA eigenvectors	2	3	11	1	1
Pseudo-R^2^	0.06	0.06	0.22	0.03	0.03
# NSA eigenvectors	1	2	1	1	2
Marginal Pseudo-R^2^	0.02	0.05	0.00	0.07	0.06
Dispersion parameter	0.31	0.38	0.32	1.55	1.24
Deviance	1.15	1.17	1.19	1.24	1.24
*Tallahassee (n = 233)*					
# PSA eigenvectors	5	1	0	2	0
Pseudo-R^2^	0.15	0.04	0.00	0.21	0.00
# NSA eigenvectors	4	0	1	0	0
Marginal Pseudo-R^2^	0.11	0.00	0.04	0.00	0.00
Dispersion parameter	0.67	1.42	0.92	3.92	6.28
Deviance	1.24	1.24	1.21	1.17	1.02
*Tampa (n = 2006)*					
# PSA eigenvectors	26	22	74	33	3
Pseudo-R^2^	0.08	0.09	0.23	0.10	0.01
# NSA eigenvectors	32	20	45	6	3
Marginal Pseudo-R^2^	0.09	0.07	0.09	0.04	0.03
Dispersion parameter	0.31	0.55	0.32	1.85	1.76
Deviance	1.15	1.19	1.19	1.25	1.23

NOTE: ^a^ An unstable estimate; bold denotes statistically significant; # denotes the number of eigenvectors.

**Table 7:** Approximate effective geographic sample size, n*.

Cancer Type	Jacksonville	Miami	Orlando	Pensacola	Tallahassee	Tampa
*n* (# block groups)	699	3377	833	267	233	2006
female breast	503	2803	450	246	172	1665
colorectal	559	2972	575	238	224	1685
lung & bronchus	447	3107	525	208	224	1364
melanoma skin	580	3276	658	240	184	1725
urinary bladder	664	3073	783	243	233	1926

NOTE: # denotes the number of observations.

## Data Availability

Restrictions apply to the availability of the cancer cases data. These data, housed in the Florida Cancer Registry, were obtained from the Florida Department of Health, and are available from that state governmental organization only after approval by its Institutional Review Board and appropriate responsible government officials.
